# Synergistic Activity of Colistin in Combination with Clofoctol against Colistin Resistant Gram-Negative Pathogens

**DOI:** 10.1128/spectrum.04275-22

**Published:** 2023-02-21

**Authors:** Diletta Collalto, Alessandra Fortuna, Paolo Visca, Francesco Imperi, Giordano Rampioni, Livia Leoni

**Affiliations:** a Department of Science, University Roma Tre, Rome, Italy; b IRCCS Fondazione Santa Lucia, Rome, Italy; University of California, San Diego

**Keywords:** antibiotic resistance, adjuvants, colistin, clofoctol, *Pseudomonas aeruginosa*, *Acinetobacter baumannii*, *Klebsiella pneumoniae*, Gram-negative pathogens, antibiotic adjuvants, pulmonary infection

## Abstract

Colistin is a bactericidal antibiotic identified decades ago which is active against a number of Gram-negative pathogens. After early elimination from clinical use due to toxicity issues, colistin has been reintroduced as a last-resort treatment for antibiotic-resistant Gram-negative infections lacking other therapeutic options. Inevitably, colistin resistance has emerged among clinical isolates, making the development of colistin adjuvants extremely beneficial. Clofoctol is a synthetic antibiotic active against Gram-positive bacteria, with low toxicity and high tropism for the airways. Interestingly, clofoctol has been found to have multiple biological activities and has been proposed for the treatment of several obstructive lung diseases, including asthma, lung cancer, and SARS-CoV-2 infection. In this study, the activity of clofoctol as a colistin adjuvant was investigated in Gram-negative lung pathogens that are critical for the high prevalence of multidrug-resistant isolates, Pseudomonas aeruginosa, Klebsiella pneumoniae, and Acinetobacter baumannii. Clofoctol potentiated the bactericidal effect of colistin in all tested strains and reduced colistin MICs below the susceptibility breakpoint in nearly all colistin-resistant strains. Overall, this observation supports the development of inhaled clofoctol-colistin formulations for the treatment of difficult-to-treat airway infections caused by Gram-negative pathogens.

**IMPORTANCE** Colistin is used as a last-resort antibiotic against extensively drug-resistant Gram-negative pathogens. However, colistin resistance is on the rise. Clofoctol is an antibiotic used against Gram-positive bacteria, with low toxicity and high penetration and storage in the airways. Here, a strong synergistic activity of the colistin-clofoctol combination against colistin-resistant Pseudomonas aeruginosa, Klebsiella pneumoniae, and Acinetobacter baumannii isolates is reported, supporting the development of clofoctol-colistin formulations for the therapy of difficult-to-treat airways infections caused by these Gram-negative pathogens.

## OBSERVATION

Colistin is a polymyxin antibiotic with bactericidal activity against Gram-negative pathogens. It was dismissed from clinical use in the late 1960s due to the concomitant development of less toxic antibiotics (1). However, the rise of infections caused by extensively drug-resistant (XDR) Gram-negative bacteria forced colistin reintroduction as a last-resort drug for the treatment of infections lacking other therapeutic options (1, 2). Consequently, colistin resistance is emerging among pathogens in clinical contexts such as ventilator-associated pneumonia and cystic fibrosis (3–5). Hence, the development of adjuvants lowering colistin therapeutic dosage and restoring susceptibility in XDR strains would be highly beneficial (6).

Clofoctol (octofene) is a synthetic antibiotic mainly active against Gram-positive bacteria. Thanks to its high tropism for the airways and low toxicity in humans (7), this drug is ordinarily prescribed in Italy and France to treat common pediatric airway infections caused by Gram-positive bacteria (8). Additional to antibacterial activity, clofoctol also regulates the unfolded protein response in eukaryotic cells, a process triggered in the airways of patients with respiratory diseases and contributing to inflammation. On these bases, clofoctol has been proposed for the treatment of different obstructive lung diseases, including asthma, lung cancer, and SARS-CoV-2 infection (8). Clofoctol also inhibits the quorum sensing (QS) cell-cell communication system of Pseudomonas aeruginosa, causing virulence attenuation (9, 10). Interestingly, a colistin-adjuvant activity has recently been discovered in two inhibitors of the P. aeruginosa QS, i.e., niclosamide and furanone C-30 (11, 12), raising the question of whether clofoctol could also display this activity.

Considering the high penetration and storage of clofoctol in airway tissues, the activity of this drug as colistin adjuvant has been investigated in three Gram-negative pulmonary pathogens for which new therapeutic options are urgently needed, namely, P. aeruginosa, Klebsiella pneumoniae, and Acinetobacter baumannii (3, 13). One colistin-susceptible (CS) and three colistin-resistant (CR) strains have been analyzed for each species (Table S1 in the supplemental material).

MIC assays (14) revealed that clofoctol has no inhibitory activity against the selected strains (MIC > 1,170 μg/mL), while colistin MIC values ranged from 0.25 to 256 μg/mL (Table 1). Checkerboard assays (14) were performed to determine possible synergism between colistin and clofoctol, combined at different concentrations. A combination showing a fractional inhibitory concentration index (FICI) of ≤0.5 was considered synergistic (15). Synergism was slightly below the threshold in CS P. aeruginosa PAO1 (FICI = 0.502), while a synergistic effect was demonstrated against CS K. pneumoniae KP-MO-27 (FICI = 0.313) and A. baumannii ATCC 19606 (FICI = 0.125) (Table 1).

**TABLE 1:** Effect of the clofoctol-colistin combination on the MIC of the indicated strains

Strain	Colistin MIC (μg/mL) at clofoctol concn (μg/mL) of:								Col_clof_^*a*^ MIC	Clof_col_^*b*^ MIC	Maximum fold change^*c*^	FICI^*d*^
	0	1.14	2.28	4.56	9.13	18.25	36.5	73				
P. aeruginosa												
PAO1	1	1	0.5	0.5	0.5	0.5	0.5	0.5	0.5	2.28	2	0.502
PAO1 col^r^1	64	64	16^*e*^	4^*e*^	2^*e*^	2^*e*^	2^*e*^	2^*e*^	2	9.13	32	0.039
BG98	32	4^*e*^	1^*e*^	1^*e*^	1^*e*^	1^*e*^	1^*e*^	1^*e*^	1	2.28	32	0.033
KK27 col^r^7	256	256	64^*e*^	16^*e*^	8^*e*^	4^*e*^	4^*e*^	4^*e*^	4	18.25	64	0.031
PAO1 Δ*pqsR*	1	1	1	0.5	0.5	0.5	0.5	0.5	0.5	4.56	2	0.504
K. pneumoniae												
KP-MO-27	0.25	0.125	0.125	0.125	0.125	0.125	0.125	0.0625^*e*^	0.0625	73	4	0.313
KP-MO-5	64	32	8^*e*^	8^*e*^	4^*e*^	4^*e*^	4^*e*^	4^*e*^	4	9.13	16	0.070
KP-MO-6	32	16	4^*e*^	2^*e*^	2^*e*^	1^*e*^	1^*e*^	1^*e*^	1	18.25	32	0.047
KP-MO-25	128	8^*e*^	4^*e*^	4^*e*^	1^*e*^	1^*e*^	1^*e*^	1^*e*^	1	9.13	128	0.016
A. baumannii												
ATCC 19606	1	0.5	0.5	0.25^*e*^	0.125^*e*^	0.125^*e*^	0.125^*e*^	0.0625^*e*^	0.0625	73	16	0.125
Ab249	128	1^*e*^	0.5^*e*^	0.5^*e*^	0.5^*e*^	0.5^*e*^	0.5^*e*^	0.5^*e*^	0.5	2.28	256	0.006
Ab347	16	2^*e*^	0.5^*e*^	0.5^*e*^	0.25^*e*^	0.25^*e*^	0.25^*e*^	0.25^*e*^	0.25	9.13	64	0.023
Ab4452	32	8^*e*^	2^*e*^	0.5^*e*^	0.5^*e*^	0.5^*e*^	0.5^*e*^	0.5^*e*^	0.5	4.56	64	0.020

a Lowest MIC of colistin in combination with clofoctol (μg/mL).

b Lowest MIC of clofoctol in combination with colistin (μg/mL).

c Ratio between colistin MIC and the MIC of Col_clof_.

d Fractional inhibitory concentration index. Clofoctol had no activity against any of the strains; therefore, the MIC of clofoctol was considered 1,170 μg/mL for calculation of the FICI [FICI = (MIC Col_clof_/MIC Col) + (MIC Clof_col_/MIC Clof)] (15).

e MIC values in which colistin/clofoctol combinations showed a synergistic activity (FICI ≤ 0.5).

Interestingly, the combination showed a strong synergistic effect in all CR strains, with MIC reductions ranging from 16- to 256-fold and FICI values of ≤0.070. In particular, clofoctol reduced colistin MICs to values below the susceptibility breakpoint (16) in all CR strains except KP-MO-5 (Table 1).

**FIG 1:**
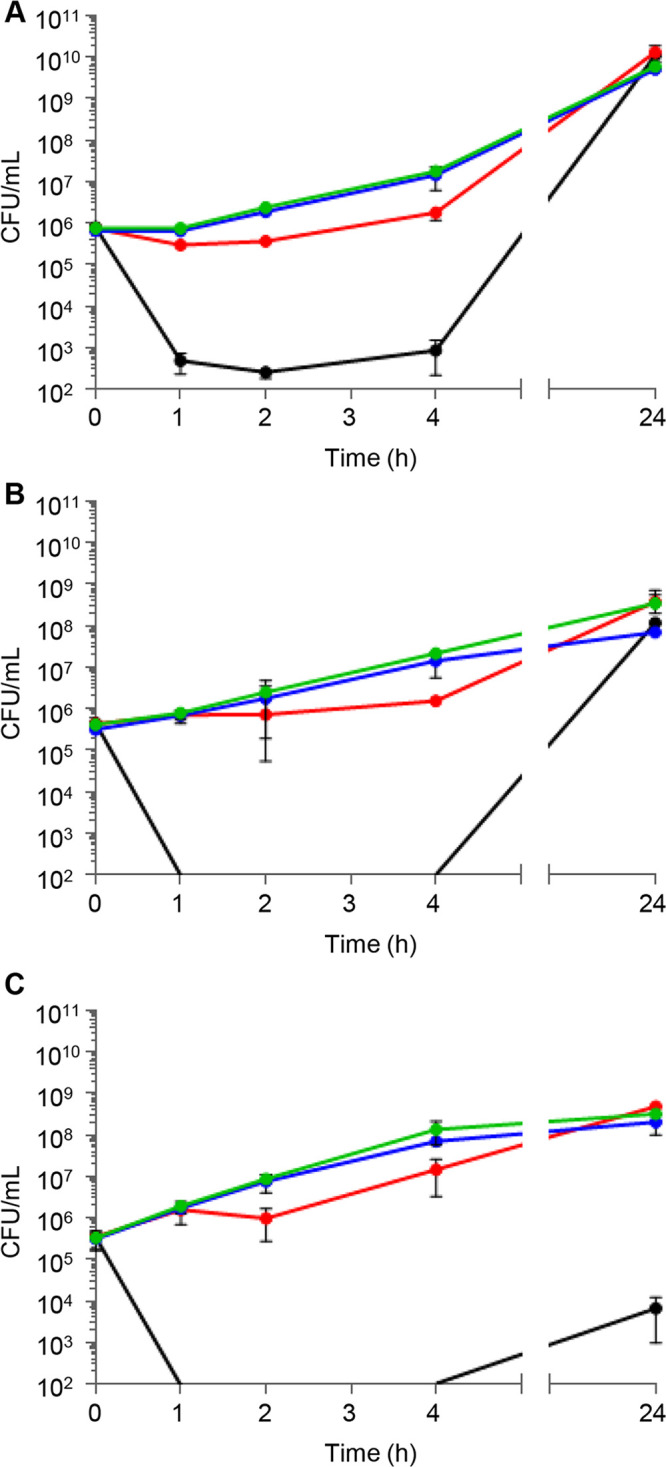
Time-kill curves of P. aeruginosa KK27 col^r^7 (A), K. pneumoniae KP-MO-5 (B), and A. baumannii Ab4452 (C) treated with 4 μg/mL colistin (red lines), 18 μg/mL clofoctol (blue lines), or the same concentrations of colistin and clofoctol in combination (black lines). The untreated controls are shown with green lines. Data are mean values from three independent experiments, and the error bars represent standard deviations. The detection limit of the assay was 10^2^ CFU/mL.

The minimal bactericidal concentration (MBC) of the combination was tested by checkerboard assays to evaluate the effect of clofoctol on colistin bactericidal activity (Table 2). In the presence of clofoctol, the MBC of colistin decreased almost in parallel with MIC reduction in all strains (MBC/MIC ratio ≤ 4), indicating that clofoctol promotes colistin-mediated bacterial killing (Table 2). Time-kill assays were performed to confirm the above-described result. One CR strain for each species was selected among the less susceptible to the colistin-clofoctol combination, namely, P. aeruginosa KK27 col^r^7, K. pneumoniae KP-MO-5, and A. baumannii Ab4452 (Table 1). Colistin and clofoctol concentrations reflecting those reached in patients’ lungs during treatment were used for this assay (17–19), i.e., colistin was tested at the minimum concentration maintained for at least 12 h in the sputum of most cystic fibrosis patients after inhaled colistin therapy (4 μg/mL) (18, 19), while clofoctol was tested at a concentration measured in the human lung 4 h after rectal administration (18 μg/mL) (17). In agreement with MBC results, the time-kill assays confirmed the bactericidal activity of the combination at colistin and clofoctol concentrations that are ineffective as monotherapy (Fig. 1), i.e., reduction of ≥3 log_10_ of the initial bacterial inoculum after 4 h of incubation. However, all cultures were able to regrow after 24 h of treatment, suggesting the emergence of resistant mutants and/or loss of activity of the combination. Further studies will be required to understand the nature of this phenomenon. Similar results were obtained with time-kill experiments carried out with the CS strains (Fig. S1).

**TABLE 2:** Effect of the clofoctol-colistin combination on the MBC of the indicated strains

Strain	Colistin MBC (μg/mL) at clofoctol concn (μg/mL)								Col_clof_^*a*^ MBC	Clof_col_^*b*^ MBC	MBC/MIC^*c*^
	0	1.14	2.28	4.56	9.13	18.25	36.5	73			
P. aeruginosa											
PAO1	4	4	2	2	1	1	1	1	1	9.13	2
PAO1 col^r^1	256	128	64	16	8	8	8	8	8	9.13	4
BG98	128	16	4	1	1	1	1	1	1	4.56	1
KK27 col^r^7	512	256	256	64	32	16	16	16	16	18.25	4
K. pneumoniae											
KP-MO-27	0.5	0.25	0.25	0.25	0.25	0.25	0.125	0.0625	0.0625	73	1
KP-MO-5	128	32	8	8	4	4	4	4	4	9.13	1
KP-MO-6	64	16	4	4	2	1	1	1	1	18.25	1
KP-MO-25	128	16	8	8	1	1	1	1	1	9.13	1
A. baumannii											
ATCC 19606	1	1	0.5	0.5	0.5	0.5	0.25	0.0625	0.0625	73	1
Ab249	128	2	2	1	1	1	1	1	1	4.56	2
Ab347	32	8	2	2	0.5	0.5	0.25	0.25	0.25	36.5	1
Ab4452	64	16	4	2	2	2	1	1	1	36.5	2

a Lowest MBC of colistin in combination with clofoctol (μg/mL).

b Lowest MBC of clofoctol in combination with colistin (μg/mL).

c Ratio between Col_clof_ MBC and Col_clof_ MIC. According to Pankey and Sabath (25), a ratio of ≤4 indicates bactericidal activity.

In P. aeruginosa, clofoctol inhibits QS by targeting the PqsR signal receptor (9). However, K. pneumoniae and A. baumannii lack the *pqs* QS system, suggesting that the clofoctol adjuvant effect could not be related to its anti-QS activity. Accordingly, P. aeruginosa PAO1 and its isogenic Δ*pqsR* mutant showed the same MIC for colistin, either alone or in combination with clofoctol (Table 1).

Since we showed that clofoctol potentiates colistin activity against bacteria characterized by different colistin resistance mechanisms (i.e., lipid A aminoarabinosylation for P. aeruginosa and K. pneumoniae, lipid A phosphoethanolamination for A. baumannii) (20–22), it is tempting to speculate that clofoctol does not interfere with specific resistance mechanisms. Unfortunately, the mechanisms underlying clofoctol antibiotic activity and bacterial resistance in Gram-positive pathogens have been poorly studied so far (8). Hence, further studies are required to confirm this hypothesis and investigate the mechanism of action of the colistin-clofoctol combination.

Overall, clofoctol synergizes with colistin against both CR and CS isolates, suggesting that it could be useful not only to treat infections caused by CR bacteria but also to reduce the colistin dosing regimen so as to minimize its toxic effects. Indeed, colistin toxicity is a concern due to its limited therapeutic range, close to the plasma nephrotoxic concentration (23). However, nebulized colistin treatment is less toxic than other administration routes, and clinical and microbiological efficacies are promising (24). Compared with other colistin adjuvants, the obvious benefit of clofoctol is that it is a commonly used antibacterial drug with known pharmacological properties, very low toxicity, and high tropism for the lungs (7, 17).

Overall, this study demonstrates that the colistin-clofoctol combination is active against relevant XDR Gram-negative pulmonary pathogens, supporting the development of inhalable clofoctol-colistin formulations.
