# Supplementary material for: Synergistic Activity of Colistin in Combination with Clofoctol against Colistin Resistant Gram-Negative Pathogens
Source: Microbiol Spectr. 2023 Feb 21;11(2):e04275-22. doi: 10.1128/spectrum.04275-22 (PMC10100712; doi:10.1128/spectrum.04275-22)
Supplement: Supplemental file 1 — Fig. S1, Table S1, and supplemental materials and methods. Download spectrum.04275-22-s0001.pdf, PDF file, 0.3 MB [file spectrum.04275-22-s0001.pdf]

Synergistic activity of colistin in combination with clofoctol against colistin resistant Gram-negative pathogens

Diletta Collalto[1], Alessandra Fortuna[1], Paolo Visca[1,2], Francesco Imperi[1,2], Giordano Rampioni[1,2], Livia Leoni[1*]

[1]Department of Science, University Roma Tre, Rome, Italy

[2]IRCCS Fondazione Santa Lucia, Rome, Italy

SUPPLEMENTAL MATERIAL

FIGURE S1. Time-kill curves of colistin susceptible strains

TABLE S1. Bacterial strains used in this study

MATERIALS AND METHODS

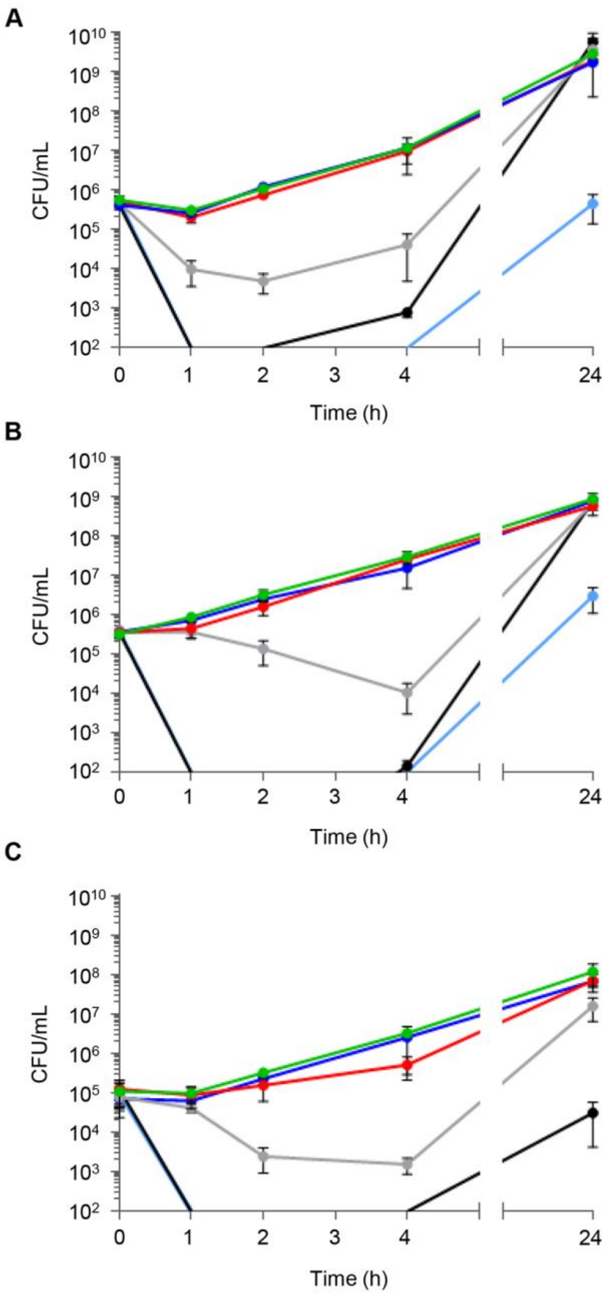

FIG S1. Time-kill curves of **(A)** *P. aeruginosa* PAO1, **(B)** *K. pneumoniae* KP-MO-27, **(C)** *A. baumannii* ATCC19606 treated with 1X MIC colistin (gray lines), 0.5X MIC colistin (red lines), 73 µg/mL clofoctol (blue lines), or the same concentrations of colistin and clofoctol in combination (black lines, 0.5X MIC colistin in combination with clofoctol; light blue lines, 1X MIC colistin in combination with clofoctol). The untreated controls are shown with green lines. **(A, B)** 1X MIC = 1 µg/mL colistin; 0.5X MIC = 0.5 µg/mL colistin. **(C)** 1X MIC = 0.25 µg/mL colistin; 0.5X MIC = 0.125 µg/mL colistin. Data are

mean values from three independent experiments and the error bars represent standard deviations

The detection limit of the assay was 10^2 CFU/mL.

TABLE S1. Bacterial strains used in this study.

Bacterial strains	Characteristics	Reference
Escherichia coli		
S17.1λ*pir*	Conjugative strain for suicide plasmids	(1)
P. aeruginosa		
PAO1 (ATCC15692)	Wild type strain, colistin-sensitive	American type culture collection
PAO1 Δ*pqsR*	PAO1-derived strain carrying an in frame clear deletion of the *pqsR* gene	This study
PAO1 col^R1	PAO1 colistin-resistant strain obtained from *in vitro* evolution experiments	(2)
KK27 col^R7	KK27 colistin-resistant strain obtained from *in vitro* evolution experiments	(3)
BG98	MDR cystic fibrosis isolate, colistin-resistant	(4)
K. pneumoniae		
KP-MO-5	Colistin-resistant clinical isolate	(5)
KP-MO-6	Colistin-resistant clinical isolate	(5)
KP-MO-25	Colistin-resistant clinical isolate	(5)
KP-MO-27	Colistin-sensitive clinical isolate	(5)
A. baumannii		
ATCC19606	Wild type strain, colistin-sensitive	(6)
Ab249	Colistin-resistant clinical isolate	(7)
Ab347	Colistin-resistant clinical isolate	(7)
Ab4452	Colistin-resistant clinical isolate	(8)

MATERIALS AND METHODS

MIC assays. MIC of colistin and clofoctol was determined with the standard broth microdilution method using Mueller-Hinton cation-adjusted broth (MH II), according to the Clinical and Laboratory Standards Institute (9). MIC was visually defined as the lowest concentration of antibiotic able to inhibit bacterial growth after 20 h incubation at 37°C under static conditions and interpreted accordingly to the EUCAST clinical breakpoints. All strains were tested in at least three independent experiments.

Checkerboard assays. Broth microdilution method was performed according to clinical laboratory standards institute (9). Briefly, colistin was 2-fold diluted in MH II along the abscissa (x-axis) and clofoctol was 2-fold diluted in MH II along the ordinate (y-axis) to test all possible combinations of the two compounds. 100 µL of a bacterial suspension containing about $5x10^5$ cells of the tested strain was added to each well. After 20 h of incubation in static conditions at 37°C, the MIC was defined as the lowest concentration of the antibiotic-clofoctol combination showing no visible growth of bacteria. All strains were tested in at least three independent experiments.

MBC assays. The Minimum Bactericidal Concentration (MBC) was obtained as follows: from the checkerboard assays, after MIC determination aliquots of 10 µL from wells where growth was inhibited were spotted on MH II agar plates and incubated for 18 h at 37°C. MBC was defined as the lowest concentration of an antibiotic required to kill 99.9% (3 log_{10}) of the starting bacterial population, *i.e.*, about $5x10^5$ cells (10). All strains were tested in at least three independent experiments.

Time-kill assays. Overnight cultures in MH II were adjusted to about $5x10^5$ cells in the same medium in the presence of colistin alone, clofoctol alone, or a combination of both (details of concentrations used are described in Fig. 1 and Fig. S1). Bacterial cultures were incubated at 37°C with shaking (200 rpm); at different time points (0 h, 1 h, 2 h, 4 h, 24 h) 100-µL aliquots were diluted serially in MH II, plated on MH II agar plates, and incubated for 18 h at room temperature before counting the number of CFU/mL. Strains were tested in at least three independent experiments.

Generation of the PAO1 Δ*pqsR* deletion mutant. The strain PAO1 Δ*pqsR* was generated by standard stepwise allelic exchange using the pDM4-derivative plasmid as previously described (11, 12). Briefly, about 500 base pairs (bp) upstream and downstream of the *pqsR* gene were PCR amplified from *P. aeruginosa* PAO1 genome with the following primer pairs: FW*pqsR*UP (5'-TGCTCTAGAACAAAAGACATAGGTTTCGGT-3') and RV*pqsR*UP (5'-CCGGAATTCAATAGGCATCCCTTATTCCTTT-3'), FW*pqsR*DOWN (5'-

CCGGAATTCGCCTGGCAACCGAGCATC-3') and RV*pqsR*DOWN (5'-ACGCGTCGACCCGTTGCCGACGATCCAG-3') (restriction sites are underlined). Resulting amplicons were cloned together in pDM4 and then conjugated from *E. coli* to *P. aeruginosa* PAO1. *P. aeruginosa* clones with a chromosomal insertion of the pDM4-derivative plasmid were selected on LB agar plates supplemented with 375 µg/mL chloramphenicol and 15 µg/mL nalidixic acid. Plasmid excision from the chromosome was subsequently selected on LB agar plates supplemented with 10% (w/v) sucrose. Deletion was checked by PCR analysis.

References (additional to those included in the main text)

1. Simon R, Priefer U, Puhler A. 1983. A broad host range mobilization system for in *vivo* genetic-engineering: transposon mutagenesis in Gram-negative bacteria. Biotechnology 1:784–791.
2. Lo Sciuto A, Cervoni M, Stefanelli R, Mancone C, Imperi F. 2020. Effect of lipid A aminoarabinosylation on *Pseudomonas aeruginosa* colistin resistance and fitness. Int J Antimicrob Agents 55:105957.
3. Lo Sciuto A, Imperi F. 2018. Aminoarabinosylation of lipid A is critical for the development of colistin resistance in *Pseudomonas aeruginosa*. Antimicrob Agents Chemother 62:e01820–17.
4. Imperi F, Fiscarelli EV, Visaggio D, Leoni L, Visca P. 2019. Activity and impact on resistance development of two antivirulence fluoropyrimidine drugs in *Pseudomonas aeruginosa*. Front Cell Infect Microbiol 9:49.
5. Esposito EP, Cervoni M, Bernardo M, Crivaro V, Cuccurullo S, Imperi F, Zarrilli R. 2018. Molecular epidemiology and virulence profiles of colistin-resistant *Klebsiella pneumoniae* blood isolates from the hospital agency "Ospedale dei Colli," Naples, Italy. Front in Microbiol 9:1–11.
6. Janssen P, Maquelin K, Coopman R, Tjernberg I, Bouvet P, Kersters K, Dijkshoorn L. 1997. Discrimination of *Acinetobacter* genomic species by AFLP fingerprinting. Int J Syst Bacteriol 47:1179–1187.
7. Pournaras S, Poulou A, Dafopoulou K, Chabane YN, Kristo I, Makris D, Hardouin J, Cosette P, Tsakris A, Dé E. 2014. Growth retardation, reduced invasiveness, and impaired colistin-

mediated cell death associated with colistin resistance development in *Acinetobacter baumannii*. Antimicrob Agents Chemother 58:828–832.

8. Durante-Mangoni E, Del Franco M, Andini R, Bernardo M, Giannouli M, Zarrilli R. 2015. Emergence of colistin resistance without loss of fitness and virulence after prolonged colistin administration in a patient with extensively drug-resistant *Acinetobacter baumannii*. Diagn Microb Infect Dis 82:222–226.
9. Clinical and Laboratory Standards Institute. 2021. Performance standards for antimicrobial susceptibility testing. M100. 31st edition. CLSI, Wayne, PA, USA.
10. Dafale NA, Semwal UP, Rajput RK, Singh GN. 2016. Selection of appropriate analytical tools to determine the potency and bioactivity of antibiotics and antibiotic resistance. J Pharm Anal 6:207–213.
11. Milton DL, O'Toole R, Horstedt P, Wolf-Watz H. 1996. Flagellin A is essential for the virulence of *Vibrio anguillarum*. J Bacteriol 178:1310–1319.
12. Fortuna A, Bähre H, Visca P, Rampioni G, Leoni L. 2021. The two *Pseudomonas aeruginosa* DksA stringent response proteins are largely interchangeable at the whole transcriptome level and in the control of virulence-related traits. Environ Microbiol 23:5487–5504.
